# Food for thought? Potential conflicts of interest in academic experts advising government and charities on dietary policies

**DOI:** 10.1186/s12889-016-3393-2

**Published:** 2016-08-05

**Authors:** Alex Newton, Ffion Lloyd-Williams, Helen Bromley, Simon Capewell

**Affiliations:** Department of Public Health and Policy, University of Liverpool, Whelan Building, Brownlow Hill, Liverpool, L69 3GB UK

**Keywords:** Policy, Food, Industry, Government, Conflict of interests, Declaration, Finance, Advice

## Abstract

**Background:**

A conflict of interest (CoI) can occur between public duty and private interest, in which a public official’s private-capacity interest could improperly influence the performance of their official duties and responsibilities.

The most tangible and commonly considered CoI are financial. However, CoI can also arise due to other types of influence including interpersonal relationships, career progression, or ideology. CoI thus exist in academia, business, government and non-governmental organisations.

However, public knowledge of CoI is currently limited due to a lack of information. The mechanisms of managing potential conflicts of interest also remain unclear due to a lack of guidelines.

We therefore examined the independence of academic experts and how well potential CoI are identified and addressed in four government and non-governmental organisations in the UK responsible for the development of food policy.

**Methods:**

Policy analysis. We developed an analytical framework to explore CoI in high-level UK food policy advice, using four case studies. Two government policy-making bodies: Department of Health ‘Obesity Review Group’ (ORG), ‘Scientific Advisory Committee on Nutrition’ (SACN) and two charities: ‘Action on Sugar’ (AoS), & ‘Heart of Mersey’ (HoM).

Information was obtained from publicly available sources and declarations. We developed a five point ordinal scale based upon the ideology of the Nolan Principles of Public Life. Group members were individually categorised on the ordinal ConScale from “0”, (complete independence from the food and drink industry) to “4”, (employed by the food and drink industry or a representative organisation).

**Results:**

CoI involving various industries have long been evident in policy making, academia and clinical practice. Suggested approaches for managing CoI could be categorised as “deny”, “describe”, or “diminish”.

Declared CoI were common in the ORG and SACN. 4 out of 28 ORG members were direct industry employees. In SACN 11 out of 17 members declared industry advisory roles or industry research funding. The two charities appeared to have equally strong academic expertise but fewer conflicts. No HoM members declared CoI. 5 out of 21 AoS members declared links with industry, mainly pharmaceutical companies. We were unable to obtain information on conflicts for some individuals.

**Conclusions:**

Conflicts of interest are unavoidable but potentially manageable.

Government organisations responsible for policy development and implementation must institutionalize an approach to identify (disclose) and manage (mitigate or eliminate) perceived and actual CoI to improve public confidence in government decision-making relevant to food policy.

**Electronic supplementary material:**

The online version of this article (doi:10.1186/s12889-016-3393-2) contains supplementary material, which is available to authorized users.

## Background

A conflict of interest (CoI) may occur when professional judgment or decisions concerning a primary interest may be unduly influenced by a secondary interest [1]. In relation to public officials, a conflict of interest can occur between public duty and private interest, in which the official’s private-capacity interest could improperly influence the performance of their official duties and responsibilities [2]. CoI can arise in different contexts and thus can be defined in different ways. CoI relate to financial interests, when an individual’s personal finances may be affected by a decision that he or she has a role in making. Alternatively, non-financial issues—such as personal relationships, business associations, and membership in political or other groups—might make it difficult for an individual to consider policy questions objectively. Ideological conflicts can also occur, for example a more libertarian view that emphasizes individual choice versus a concern with the broader social impact of those choices. In this instance, it is often more difficult to distinguish “conflicts of interest” that should be avoided from “policy disagreements” that should be addressed in the actions of public office [3].

A conflicted expert will not necessarily be less objective than a non-conflicted counterpart [4]. However, logical concerns are supported by extensive psychological research [5–7] plus empirical data from the tobacco, alcohol, pharmaceutical and medical device industries. Reviews, including a recent Cochrane meta-analysis [8] show that an individual with financial or other links to a company will likely favour that corporation, consciously or subconsciously [8–14]. Poor methodology and publication bias are commonplace in industry funded research [10, 14]. Industry funded literature reviews are also known to be susceptible to industry influence from CoI [9, 15, 16]. Between March and October 2015, Nestle identified 76 industry funded food and nutrition studies. Of these 70 reported results favourable to the sponsors [14].

The National Audit Office state that “departments and agencies should have a code of ethics or code of conduct. These standards should define what behaviours and practices are acceptable and unacceptable, and should clearly state what will happen when people do not comply.” They also raise the issue of the potential risk of a legal challenge to decisions made by public bodies. If a decision-maker has a conflict of interest then the decision is potentially vulnerable and could be overturned on judicial review [17]. CoI are concerning and should be examined in order to ensure objectivity is maintained and those in public office are open to scrutiny. Identifying and preventing or resolving CoI situations is crucial to good governance and maintaining trust in government decisions and policy.

Applying the CoI label does not imply that the conflicted expert is necessarily guilty of collusion or corruption. However, current or past financial or personal associations with interested parties make it difficult to distinguish subtle, unconscious bias from deliberately concealed impropriety. Being ‘conflicted’ thus also means ‘potentially conflicted’, as the term can be applied before key events, such as decision-making or research publication. Perceived CoI are also important, potentially tarnishing the reputation of scientists, organisations or corporations [2, 17, 18]. This then offers scope for preventive measures.

In the UK, The Nolan Principles of Public Life [19] (Table 1) demand standards of professional conduct and integrity for public officials. In use since 1995, these principles aim to combat the risks (bias towards financial, business/industry interests), that CoI pose to procedures conducted for the public good. Neglecting the application of these principles could foreseeably cause a great number of problems, in terms of objectivity and accountability, both politically and for evidence-based public health. However, it is less clear whether central UK government routinely follows these principles when eliciting advice on food policy.

**Table 1:** The Nolan principles of public life

Principle	Explanation
Selflessness	Holders of public office should act solely in terms of the public interest. They should not do so in order to gain financial or other benefits for themselves, their family or their friends
Integrity	Holders of public office should not place themselves under any financial or other obligation to outside individuals or organisations that might seek to influence them in the performance of their official duties
Objectivity	In carrying out public business, including making public appointments, awarding contracts, or recommending individuals for rewards and benefits, holders of public office should make choices on merit
Accountability	Holders of public office are accountable for their decisions and actions to the public and must submit themselves to whatever scrutiny is appropriate to their office
Openness	Holders of public office should be as open as possible about all the decisions and actions that they take. They should give reasons for their decisions and restrict information only when the wider public interest clearly demands
Honesty	Holders of public office have a duty to declare any private interests relating to their public duties and to take steps to resolve any conflicts arising in a way that protects the public interest
Leadership	Holders of public office should promote and support these principles by leadership and example

**Table 2:** Proposed scoring system (ConScores) for members of advisory groups

ConScore				
0 (100 % Independent of industry)	1	2	3	4 (Industry Employee)
Zero interaction				
	Received hospitality	Received hospitality	Received hospitality	Received hospitality
		Research Funding	Research Funding	Research Funding
			Consultancy	Consultancy
			Industry Shareholder	Industry Shareholder
				Employed by food company
				Employed by organisation representing industry

**Table 3:** Proposed typology of management of conflicts of interest

Standpoint	Suggested management	Number of studies recommending suggested management	%
Deny	Do Nothing	4	15
	Increase Self-Regulation and/or Professional Standards	11	42
Describe	Education on CoI	5	19
	Improve Transparency	21	81
	Introduce Central Repository for industry Funding	3	12
	Standard CoI Policy Across Multiple Centres/Disciplines	4	15
Diminish	Prevent/Limit Industry Interaction	15	58
	Independent Non-Conflicted Group to Review CoI	11	42
	Sanctions for Those Who Breach Policy	2	8

Public health policy in England comes mainly from the Department of Health (DH). The DH has successfully implemented strategies on tobacco control, alcohol, physical activity and food [20, 21]. Currently, high rates of obesity and diabetes in adults and children in the UK have recently fuelled increasing concern about sugar intake [22–24]. Government food policy is now under increasing scrutiny [13].

However, any regulation or tax on sugar would potentially threaten transnational corporations’ sales and profits. Industry opposition might therefore be predicted using a variety of denialism tactics, including influencing scientific researchers, and hence the expert advice given to policy makers [8].

Gornell uncovered a “tangled web” of connections between the sugar industry and UK government advisory bodies with key public health experts involved with the sugar industry and related companies responsible for many of the products blamed for the obesity crisis through research grants, consultancy fees, and other forms of funding [25]. Some of the experts whose reputations were publicly questioned subsequently responded to defend these allegations [26], however the concern is that a CoI may bias behaviour, and it is the potential for bias that makes CoI important [27].

Many governments are now emphasizing public/private partnerships [28–30], resulting in closer ties to industry. Subsequent CoI are becoming increasingly common within decision-making groups. By influencing the academic evidence base and subsequent discussion in policy making groups, powerful corporations with vested interests and considerable public, political and media influence are well positioned to lobby governments. This can cause governments to base policy not on nutrition and public health effects of their products but on wealth creation and the financial and employment potential of their companies.

The normalisation of public/private partnerships has generally fostered a relaxed attitude towards the impact of CoI [31]. Indeed, many at risk individuals claim that CoI are unlikely to cause problems [32, 33]. Academics aspire to be independent and objective, but they are also human, and therefore vulnerable to potential CoI.

Adams (2007) suggests the concept of a ‘continuum’ of moral jeopardy is preferable to a ‘binary’ interpretation, [34] with a scale ranging from those with minor involvements to those with unmanageable conflicts of interest. He suggests active scrutiny of these risks can be assisted through strategies that promote ongoing self-assessment [34]. Many academics assert that their academic integrity and self-assessment will nullify any potential bias from commercial CoI [35]. However, the evidence shows otherwise. CoI have been consistently linked to biased outcomes, which compromise the quality of academic evidence and undermine supposedly ‘evidence based’ decisions [11–14].

Although the full influence of industry is not known, it is possible that high-level food policy decisions in the UK may be affected by corporate attempts to influence policy. This has certainly been highlighted in papers by Moodie [8], Stuckler [36] and most recently by Gornall [25].

However, public knowledge of CoI is currently limited due to a lack of information and the complexity of this issue. The mechanisms for identifying and managing potential conflicts of interest also remain unclear [2, 17, 18].

We therefore examined four contrasting governmental and non-governmental organisations in the UK responsible for developing food policy based on current research (The Department of Health ‘Obesity Review Group’ (ORG), The ‘Scientific Advisory Committee on Nutrition’ (SACN) and two charities: ‘Action on Sugar’ (AoS), and ‘Heart of Mersey’ (HoM) (Additional file 1: Appendices 1 - 6). All four use academic topic experts to give advice and help formulate public policy. We asked:

*How independent of industry influence are these academic “experts” and how well are potential conflicts of interest identified and addressed?*

## Methods

We examined potential CoI in four case studies: two government policy-making bodies and, as possible exemplars, two charities, one food-based and one promoting cardiovascular health:

1. The ‘Obesity Review Group’ (ORG), an arm’s length advisory group to the DH.
2. The Scientific Advisory Committee on Nutrition (SACN), a committee advising Public Health England and the government on food matters.
3. ‘Action on Sugar’ (AoS), a non-governmental public interest campaigning charity.
4. ‘Heart of Mersey’ (HoM), an independent regional cardiovascular health charity.

All groups were chosen from the UK because all authors are based in the UK and are therefore most familiar with UK public health groups/structures and methods of policy generation. As there are relatively few leading organisations specifically involved in UK food policy there were not many options to choose from. We hoped to maximise contrast between groups in our analysis, using two which are close to government and two non-governmental, third sector organisations. We therefore chose ORG, SACN, AoS and HoM: a governmental organisation, an organisation ‘at arm’s-length’ from the government plus a national and a regional charity.

The membership of each advisory group was determined via the organisation website, with initial data on potential conflicts being obtained through personal biographies on organisation and employer websites or networking sites such as LinkedIn.

CoI declarations were identified through systematic Google and Google Scholar searches. Sources included publicly accessible CoI records, minutes of public office meetings and academic publications. CoI declarations used in this analysis are detailed in Additional file 1: Appendices 2 - 7.

Data were collated in a Microsoft Excel database of CoI relating to food policy. Only ‘recent’ CoI declarations (last 5 years) were included. Institutions differ on the length of time for which past interests must be declared, usually between 1-10 years depending on the nature of the conflict and reason for declaration.

Each member of each group was then categorised on a five point ordinal ConScale (Table 2). The ConScale was conceived and trialled by the authors as a method of differentiating between levels of conflict in individuals. Scores range from “0”, (indicating complete independence from the food and drink industry) to “4”, (an employee of the food and drink industry or representative organisation) or ‘unknown’ if there was a complete absence of CoI declarations for the individual.

Individuals score “0” if all declarations report no CoI. A score of “1” represents receipt of industry hospitality. ‘Hospitality’ can cover a broad spectrum in CoI declarations, but is taken to mean expenses - accommodation, meals, travel or entertainment – beyond what is reasonably required [37]. In CoI declarations it is routine to declare ‘hospitality’, without differentiating further. We therefore considered it suitable to do the same in our analysis.

A score of “2” is given for receipt of funding for research. Acting in a consultancy role or being a shareholder in a relevant industry scores “3”. The highest score of “4” is reserved for individuals primarily employed by industry or representing the commercial interests of industry.

Stronger links to industry indicated by higher scores increase the dependence of the individual on the corporation and increases a conscious or sub-conscious supposition of reciprocity to those who have benefitted them. Levels of dependence and reciprocity may vary circumstantially. Therefore, the level of perceived conflict and moral questionability of interacting with industry can change accordingly along a spectrum. The impact of a conflict due to research funding may in some cases be much greater than that of a conflict due to consultancy. Equally, some indirect CoI may have more impact than direct conflicts. However, any overarching classification system is not able to take account of such variability. Importantly, the level of conflict perceived by the public is unlikely to alter due to such circumstances.

## Results

### Four case studies in UK food policy (Fig. 1) (Additional file 1: Appendices 2 - 6)

**Fig. 1:**
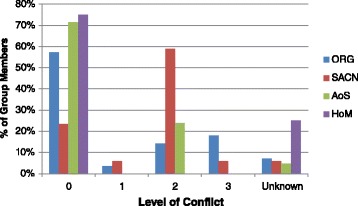
Conflicts of interest within advisory groups

There was surprisingly little information publicly available on the interests of members in the four bodies examined. This was particularly evident for non-academic members. Data were often difficult to find, opaque and hence difficult to interpret. Each group contained at least one member with no publicly available CoI declarations.

Declared CoI were common in the Obesity Review Group (ORG). Four out of 28 of the members were direct employees of industry. Five members were paid consultants for the food and drink industry, of which one was also an industry shareholder. Two members had an unknown level of conflict. Furthermore, there are no records of CoI declarations published by the ORG or evidence of declarations of interest within the minutes of meetings [38, 39].

The Scientific Advisory Committee on Nutrition (SACN) hold full CoI declarations for all members which are published in their annual report, part of an extensive, publicly accessible catalogue of reports and meeting minutes. However, 9 out of 17 SACN members declared advisory roles for industry and 2 out of 17 members declared receiving research funding from industry. The group also includes 4 industry shareholders and one member is primarily employed as a ‘science leader’ in the research and development department at Unilever. One member, the lay member of the group, had no identifiable declarations of interest. Neither of the two charities had any members employed directly by industry. Five of 21 members of Action on Sugar (AoS), declared financial links with industry. Three of these were related to pharmaceutical industries, two who were paid for past consultancy work and one member who was categorised as conflicted due to holding patents for a medication to prevent cardiovascular disease. One AoS member had no publicly available CoI declarations. No Heart of Mersey (HoM) members declared any ties to industry, although 3 out of 12 members had no publicly available declarations. HoM explicitly claim adherence to the Nolan Principles of Public Life (Table 3).

## Discussion

Based on our rapid literature review (Additional file 1: Appendix 8), approaches for managing conflicts of interest could be categorised as either ‘deny’, (ignore or conceal) ‘describe’ (document then dismiss) or ‘diminish’ (actively identify, minimise or prevent) (Table 3). These categories can be applied to the four case studies. We suggest that this analytical framework of categorising approaches to managing CoI might be useful in future studies and practice.

Despite repeated calls for transparency, there was surprisingly little information available on the ORG members’ interests. Even with the limited information found, many members were conflicted, including four individuals directly employed by industry. It follows that the ORG approach to CoI is to ‘deny’, that is to conceal or ignore any existing CoI.

SACN are more transparent, thus more revealing, clearly demonstrating a ‘describe’ strategy. Commendably, the group records and publishes declarations of interest by group members annually and minutes of SACN meetings are also published online. Analysis of the minutes from the last 5 years show that attendees are routinely obliged to declare any new conflicts at the start of each meeting. However, there is scant mention of action taken to remove conflicts from decision making processes other than a retroactive statement following a negative media reporting of potential conflicts within the group (Additional file 1: Appendix 9).

AoS and particularly HoM comprise notably fewer conflicted individuals. It is possible that the AoS Chair felt that links to the pharmaceutical industry were unlikely to conflict advice on sugary drinks or processed foods. HoM in particular appear to demonstrate that comparable experts with minimal CoI exist and are available.

Both charities appear to make some efforts to ‘diminish’ CoI while maintaining equally strong or even greater academic expertise. However, both of these groups are less procedurally transparent than SACN. This should be improved to prove a commitment to actively identifying, minimising and preventing CoI.

The ORG and SACN are public health bodies focussed on improving population health by advising on nutrition and health related issues. Both therefore need to command total public confidence in their procedures as well as their products. Although a “Describe” strategy might be better than a “Deny” approach, neither provide adequate public safeguards. Merely documenting an individual’s widespread industrial interests is neither convincing nor adequate. To then assert that “no further action” is required clearly undermines the perceived objectivity of the expert advice and erodes public confidence.

Prescriptive mechanisms to “Diminish” conflicts are clearly required if ORG and SACN wish to manage CoI effectively and convincingly. Current practice is thus falling far short of the strong recommendations made in the literature.

The Nolan Principles (Table 1) potentially provide a practical and effective solution to help policy makers actively manage any CoI, whether current or perceived. These Principles are increasingly seen as good practice models for management of CoI [40]. Specifically, their application would exclude academic experts associated with industry funding on any policy advisory panel on the basis of “Selflessness”, “Integrity”, and “Objectivity”. Yet, where these principles should be followed most stringently, within the UK government, they appear to be overlooked.

Media exposure of professionals who accept funding to support nutrition research is increasing government pressure for UK reform to address CoI [25, 41]. Internationally, there is also increasing unease over the influence of powerful corporations. Founded in 2011, the ‘Conflicts of Interest Coalition’, representing over 160 regional, national and international networks and organisations united by the common objective of safeguarding public health policy-making against commercial conflicts of interest through the development of a Code of Conduct and Ethical Framework for interactions with the private sector, released a ‘Statement of Concern’ [42]. The coalition has called on the World Health Organization (WHO) to further ‘safeguard public health policy from commercial influence’ [43] and to ‘stop blurring the distinctions between actors who share the primary interest with WHO and those whose primary interest is market-led’ [44]. In response to this call, WHO are now in the process of finalising a ‘Framework of engagement with non-State actors’ [45].

Yet, the recent UK government, like some other countries [46, 47] has increased industry involvement in policy making through public private partnerships and the ‘Responsibility Deal’ [48]. Concerns about the nature of these relationships have been raised but ignored [49–52]. Yet such partnership approaches and voluntary agreements enable corporations, whose primary interest is profit, to dilute or delay more effective policies implemented by government to promote public health, such as regulation or taxation [48, 49, 53].

In October 2015 the WHO convened a technical consultation to address and manage conflicts of interest in the planning and delivery of nutrition programmes at the country level. The consultation paper recommended that institutions should emphasize the prevention of CoI rather than their management. The paper offers proposed indicators for a variety of topics including obesity and NCDs, and recommends several pre-requisites prior to government or non-governmental organisations engaging with private-sector actors who may unduly influence food and nutrition policy processes and outcomes. Furthermore, the WHO consultation enabled ideologically diverse actors to come together and help lead to reciprocity to foster an enabling environment for CoI to evolve.

There are various publications in the grey literature which attempt to address the strengths and weaknesses of existing approaches to identify, manage and mitigate CoI. These publications provide valuable guidance for preventing and addressing CoI, whilst at the same time demonstrating the complexities involved for individuals and organisations in ensuring impartiality [2, 17, 18, 54].

Our study indicates that conflicts of interest exist in organisations directly involved with influencing UK food policy. Declared CoI were common in both UK government food policy bodies reviewed, the Scientific Advisory Body on Nutrition (SACN) and the Obesity Review Group (ORG). Both SACN and the ORG include individuals directly employed by the food industry.

### Limitations

Perhaps the main limitation of the case studies was the patchy and probably incomplete data on individuals’ interests in the four bodies analysed. Reporting standards were very variable. Some individuals, particularly those outside of academia, had no publicly searchable information whatsoever. CoI declarations for academics were more accessible but still neither exhaustive nor consistent. Furthermore, we did not contact individual members of each group or conduct systematic Freedom of Information requests. Under estimation of CoI is thus likely.

We created and piloted a five point ordinal ConScale from “0”, (indicating complete independence from the food and drink industry) to “4”, (an employee of the food and drink industry or a representative organisation). While manifesting good face validity, more formal evaluation and validation is now indicated.

Due to limitations of time and resources we were restricted to documenting potential CoI rather than determining their impact on food policy. Further research would benefit from using the ConScale to explore the relationship between the organisation’s individual and aggregate CoI scores and the decisions that were reached.

All four case studies examined UK-based food policy influencers. Future analyses might consider analysing larger numbers of diverse organisations from a variety of high middle and low income countries [36].

### Implications for further research and for future health policy

This exploratory study highlights some key issues surrounding CoI in policy making and thus identifies important topics for future research. This study demonstrates an urgent need to increase the availability of data to assure public confidence in the integrity of policy making groups. There is potential value in a mandatory public register of public servants’ CoI declarations, a strategy strongly promoted within the literature.

The scarcity of literature regarding CoI in policy makers was surprising. We found none specifically concerning academic experts advising on food policy. Future projects might examine a wider range of groups that influence public health policy making. Ideally these investigations would all be reinforced by Freedom of Information requests followed by invitations enabling each individual group member to comment. The “National Academies of Sciences, Engineering and Medicine, US” model recently adapted by NICE requiring a neutral chair [55] might also be formally evaluated. While this analysis is limited to UK organisations, the principles of this study and the implications outlined above may be cautiously generalizable elsewhere.

## Conclusions

Conflicts of interest are unavoidable if organisations wish to enlist a representative spectrum of experts to advise on evidence-based policies. However, they are potentially manageable if guidelines such as The Nolan Principles of Public Life are adhered to. Government organisations responsible for policy development and implementation must institutionalize an approach to identify (disclose) and manage (mitigate or ideally eliminate) perceived and actual CoI to improve public confidence in government decision-making relevant to food policy.

## Abbreviations

AoS, Action on Sugar; CoI, conflict of interest; HoM, Heart of Mersey; NICE, National Institute for Health and Care Excellence; ORG, Obesity Review Group; SACN, Scientific Advisory Committee on Nutrition; UK, United Kingdom; WHO, World Health Organization

## Additional file

Additional file 1: Food for thought? Potential conflicts of interest in academic experts advising government and charities on dietary policies. **Appendix 1:** Description of ORG, SACN, AoS and HoM. **Appendix 2:** Obesity Review Group (ORG): Publicly Available Declarations of Interest. **Appendix 3:** Scientific Advisory Committee on Nutrition (SACN): Publicly Available Declarations of Interest. **Appendix 4:** Action on Sugar (AoS): Publicly Available Declarations of Interest. **Appendix 5:** Heart of Mersey (HoM): Publicity available Declarations of Interest. **Appendix 6:** Sources for Declarations of Interest: ORG, SACN, AoS and HoM. **Appendix 7:** Identifying publicly accessible CoI declarations. **Appendix 8:** Rapid Literature Review. **Appendix 9:** Analysis of SACN meeting minutes. (DOCX 108 kb)
