# Supplementary material for: Food for thought? Potential conflicts of interest in academic experts advising government and charities on dietary policies
Source: BMC Public Health. 2016 Aug 5;16:735. doi: 10.1186/s12889-016-3393-2 (PMC4975877; doi:10.1186/s12889-016-3393-2)
Supplement: Additional file 1: — Food for thought? Potential conflicts of interest in academic experts advising government and charities on dietary policies. Appendix 1: Description of ORG, SACN, AoS and HoM. Appendix 2: Obesity Review Group (ORG): Publicly Available Declarations of Interest. Appendix 3: Scientific Advisory Committee on Nutrition (SACN): Publicly Available Declarations of Interest. Appendix 4: Action on Sugar (AoS): Publicly Available Declarations of Interest. Appendix 5: Heart of Mersey (HoM): Publicity available Declarations of Interest. Appendix 6: Sources for Declarations of Interest: ORG, SACN, AoS and HoM. Appendix 7: Identifying publicly accessible CoI declarations. Appendix 8: Rapid Literature Review. Appendix 9: Analysis of SACN meeting minutes. (DOCX 108 kb) [file 12889_2016_3393_MOESM1_ESM.docx]

**Food for thought? Potential conflicts of interest in academic experts advising government and charities on dietary policies**

**Appendices**

**A1 –** Description of ORG, SACN, AoS and HoM

**A2 –** Obesity Review Group (ORG): Publicly Available Declarations of Interest

**A3–** Scientific Advisory Committee on Nutrition (SACN): Publicly Available Declarations of Interest

**A4 –** Action on Sugar (AoS): Publicly Available Declarations of Interest

**A5**– Heart of Mersey (HoM): Publicity available Declarations of Interest

**A6 –** Sources for Declarations of Interest: ORG, SACN, AoS and HoM

**A7 –** Identifying publicly accessible CoI declarations

**A8 –** Rapid Literature Review

**A9 -** Analysis of SACN meeting minutes

**APPENDIX 1 Description of ORG, SACN, AoS and HoM**

**The Department of Health Obesity Review Group**

*Purpose*

The overarching purpose of the Review Group is to bring together a range of experts and delivery partners to take stock of progress in meeting national ambitions to reduce excess weight and to consider what more needs to be done to achieve maximum impact.

*The Group’s objectives are to:*

• Consider and interpret national statistics on excess weight. This will include noting annually whether England is on track to meet the national ambitions, as well as considering important patterns and advising on the factors driving these, and the implications for future priorities.

• Share and synthesise insights, evidence and experience of initiatives to address or prevent excess weight and of the wider obesity landscape, to build a robust picture of thinking on particular topical issues and highlight important new learning.

• Based on the above, advise on opportunities for research and support in the future for DH and others to consider. These might cover gaps in current knowledge, national policy, or support for local efforts.

**The Scientific Advisory Committee on Nutrition (SACN)** advises Public Health England and other government agencies and departments on nutrition and related health issues.

SACN advises on:

- nutrient content of individual foods, and on diet as a whole including the definition of a balanced diet, and the nutritional status of people
- nutritional status of people in the UK and how it may be monitored
- nutritional issues which affect wider public health policy issues including conditions where nutritional status is one of a number of risk factors (e.g. cardiovascular disease, cancer, osteoporosis and/or obesity)
- nutrition of vulnerable groups (e.g. infants and the elderly) and health inequality issues
- research requirements for the above

**Action on Sugar**

Action on Sugar is a group of specialists concerned with sugar and its effects on health. It is working to reach a consensus with the food industry and the UK Government over the harmful effects of a high sugar diet, and bring about a reduction in the amount of sugar in processed foods. 

Action on Sugar is a registered charity and is funded by charitable donations. It does not accept any funding from the food or soft drinks industry. Action on Sugar is supported by 23 global expert advisors.

**Heart of Mersey**

Heart of Mersey is based in the North West of England and was established over 10 years ago. It is a social enterprise that delivers practical, evidence-based, programmes and interventions that seek to tackle the root causes of health inequalities.

It specialises in tobacco control, diet and nutrition and physical activity and designs and delivers applied public health products that help you to improve the health of the local community.

**Appendix 2 – Obesity Review Group (ORG): Publicly Available Declarations of Interest**

| **Title & Initials** | **Employer (as listed on DoH website)** | **Other Employment/Interests** | **Significant Past Employment/Interests** | **Academic Institution** | **Rating (Based on publicly available CoI Declarations)** |
| --- | --- | --- | --- | --- | --- |
| JA | Institute of Health Equity UK | co-director of the Review of Social Determinants of Health and the Health Divide in the WHO European Region | Project Director of the Strategic Review of Health Inequalities in England post-2010 (the Marmot Review),head of Health and Social Care at IPPR, Research Fellow in Public Health at the Kings Fund, Unicef, LSE | UCL | 0 |
|
|
|
|
|
| LA | Diabetes UK | Medical Research Foundation Trustee | Head of Communications at Lambeth Council, media specialist at Which? , Food Standards Agency and Health Education Authority |  | 0 |
|
|
|
|
| PA | University of Oxford | President of the UK Society of Behavioural Medicine, trustee of the Association for the Study of Obesity, member of the Society for Research on Nicotine and Tobacco. Editor of the journal Addiction | University of Birmingham, GP, Consultancy for Xenova - a biotechnology company investigating a nicotine vaccine | Oxford | 3 |
|
|
|
|
|
| JB | Leicestershire and Rutland County Sports Partnership |  | CEO of Sport Cheshire |  | No declarations found |
|
|
|
| SB | Local Government Association | Cabinet Member for Health and Wellbeing, Birmingham City Councillor, Managing Director of FD associates - also called fairy dust consulting |  |  | 0 |
|
|
|
|
| CC | British Dietetic Association | Chief Dietician St Georges NHS hospital | Northampton General Hospital |  | No declarations found |
|
| SD | Which? | EFSA |  |  | 0 |
| CD | NHS Alliance | Newcastle Bridges GP Consortium | HEALTHWORKS Newcastle | Northumbria | 0 |
|
| KF | Public Health England |  | director of the National Center for HIV/AIDS, Viral Hepatitis, STD, and TB Prevention - part of the CDC, |  | 0 |
|
|
|
| NF | UCL Hospitals | Consultant Endocrinologist and Bariatric Physician | clinical director of the Wellcome Trust Clinical Research Facility, Shareholder in Counterweight plc, consultancy and speaker’s fees from Novo Nordisk, Janssen, Vivus. | UCL | 3 |
|
| DH | National Obesity Forum | Physician specialising in Obesity Medicine at the Centre for Obesity research at Luton & Dunstable Hospital. | member of the Counterweight Scientific Advisory Board, acts as a consultant to Lighter Life, received honorariums for presentations and advisory board attendance from Sanofi-Aventis, Abbott, Roche and GlaxoSmithKline |  | 3 |
|
|
|
| ZG | Weight Watchers Europe |  | Dietitian - Cardiff and Vale NHS |  | 4 |
|
|
| AH | University of Leeds | editorial board member of the International Journal of Obesity, Body Image, and Pediatric Obesity | Past Chairman of the UK Association for the Study of Obesity (ASO) | leeds | 0 |
|
|
| PI | Sustrans Ltd | Senior member of staff for Sustrans, a charity who work to promote walking and cycling |  |  | 0 |
| SJ | University of Oxford | Chair the DH Public Health Responsibility Deal Food Network, member of the Public Health England Obesity Programme Board, one of the Chairs of the NICE Public Health Advisory Committees | Head of Nutrition/Health MRC, research group has received research funding from Department of Health, European Union, Food Standards Agency, Medical Research Council, World Cancer Research Fund, Coca-cola, Weight Watchers.  Member of Scientific Advisory Boards for Coca-Cola, Heinz, PepsiCo, Nestlé and Kellogg's Tanita: 'In a personal capacity I am a Member of the Tanita Medical Advisory Board', receive a fee for writing articles for the Rosemary Conley Diet and Fitness magazine. | Oxford | 3 |
|
|
|
|
|
|
| ML | Food and Drink Federation | carnegie UK trust |  |  | 4 |
|
|
| PL | UK Health Forum |  | Director at the Health Education Authority, NHS, Researcher, Teacher | Southampton | 0 |
|
|
| IM | University of Nottingham | NHS ethics committee -researcher, advisory roles on; the Mars Europe Advisory Board, Coca Cola European Scientific Advisory Committee and Coca Cola International Public Policy Advisory Board, Waltham Centre for Pet Nutrition (owned by Mars), |  | Nottingham | 3 |
|
|
|
|
|
|
|
| MM | Sport and Exercise Sciences Research Institute | Member of the UK Expert scientific panel for the UK Physical Activity Guidelines, Director of the Sports Institute for Northern Ireland, Director of the City of Lisburn Salto National Gymnastics Centre | Member of the Sports Council for Northern Ireland , Member of the General Teaching Council for Northern Ireland, Development Director for Gymnastics Northern Ireland | Ulster | 0 |
|
|
| AO | British Retail Consortium |  |  |  | 4 |
|
| SP | Alliance Healthcare |  |  |  | 4 |
|
| DR | Director of Public Health, Rotherham | Advises NICE technology appraisals on wide variety of areas | GP, Sheffield University (Examiner) |  | 0 |
|
|
| AR | Director of Public Health, Greater Manchester |  | Lecturer at Liverpool JMU, Liverpool Central PCT |  | 0 |
|
|
|
| MS | Ealing Council |  | Chief Executive of Tower Hamlets Council |  | 0 |
|
| TS | Academy of Medical Royal Colleges | President of the Royal College of Paediatrics and Child Health, Professor of Child Health at the Institute of Child Health, University College London | Dean of the Medical School and Professor of Child Health at the University of Nottingham, Non-Executive Director of Nottingham University Hospitals NHS Trust | UCL | 0 |
|
|
|
|
| JV | NHS England (clinical director for obesity/diabetes) | Consultant Diabetologist, Physician and Endocrinologist at Imperial College Healthcare NHS Trust, Adjunct Professor in Diabetes and Endocrinology at Imperial College London, |  | Imperial College | 0 |
|
|
|
|
|
| JW | Weight Concern | Professor of Clinical Psychology Epidemiology & Public Health and Director of the Cancer Research UK Health Behaviour Research Centre at UCL, founding editor of the British Journal of Health Psychology, is on the editorial board of 8 international journals | Imperial Cancer Research Fund (ICRF) Health Behaviour Unit and Reader in clinical psychology at the Institute of Psychiatry | UCL | 0 |
|
|
|
|
|
|
|
| CG | Royal College of Midwives | Practicing Midwife Won award for helping pregnant women with obesity and related issues | Recognised by NICE for her part in developing shared learning database and commissioning guide for Public Health Guidance |  | 0 |
|
|
|

**Appendix 3 –** **Scientific Advisory Committee on Nutrition (SACN): Publicly Available Declarations of Interest**

| **Title & Initials** | **Employer (as listed on SACN webiste)** | **Other Employment/Interests** | **Significant Past Employment/Interests** | **Academic Institution** | **Rating (Based on publicly available CoI Declarations)** |
| --- | --- | --- | --- | --- | --- |
| AP | Director of MRC for human nutrition | Research funding from World Cancer Research Fund Conference dnation from National Centre for Science Research. IP exploitation for The Boden Institute of Obesity . Academic centre takes research funding from: Action Medical Research, Coca Cola, Diabetes UK, Nestle, The Institute of Brewing and Distilling, Thermo Fisher Scientific, Weight Watchers, Conference donations from: Electro Sci Industries Iron Therapeutics, Switzerland AG, Kelloggs, LLR-G5, Playerthree, British Dietetic Association. Consultancy work for Shield Holding. | Former president of Nutrition Society, | Cambridge (MRC) | 3 |
| PA | Honorary Professor, Lancaster University | chair of the Standing Committee on Nutrition for the Royal College of Paediatrics and Child Health. Consultancy and research for New Zealand Dairy Goat Council. Chaiman and receives lecture fees from International Association of Dietary Supplements Associations ILSI | past chair and secretary of the European Society for Paediatric Gastroenterology, Hepatology and Nutrition’s Committee on Nutrition, Served on national and international advisory committees relating to clinical nutrition, public health and preventative medicine. | Lancaster | 3 |
| PH | Head of Nutrition & Epigenetics and Senior Lecturer at Rowett Institute of Nutrition and Health,UniversityofAberdeen | Honarary Clinical Scientist in Grampian NHS Trust, member of the Advisory Committee on Novel Foods and the North of Scotland Research Ethics committee. Member of the Breast Cancer Campaign Scientific Advisory Board, Recieved reviewing fees from FSA which contributed to research funding. Shareholder, Cafe Direct | Lecturer at Liverpool JMU, Liverpool Central PCT | Aberdeen | 3 |
| TK | Deputy Director, Cancer Epidemiology Unit,University of Oxford | lead investigator of theOxford component of the European Prospective Investigation into Cancer (EPIC), chair of the EPIC Working Group on Nutrition and Prostate Cancer, and chair of the Endogenous Hormones and Breast Cancer Collaborative Group |  | Oxford | 0 |
| DM | Science Leader at Unilever R&D Vlaardingen | Employee and Shareholder of Unilever | academic research career in theUSandUK |  | 4 |
| IM | Professor of Metabolic Physiology at the University ofNottinghamand Director of Research in the Faculty of Medicine and Health Sciences | President of the Nutrition Society, Editor to the International Journal of Obesity and Chair of the International Association for the Study of Obesity Finance Committee. SACN representative for Responsibility Deal Food Network. Research Funding from Mars and Unilever. 'Meeting attendance' with Mars and Coca Cola. | University of Birmingham, GP, Consultancy for Pfizer, McNeil and Xenova - a biotechnology company investigating a nicotine vaccine | Nottingham | 3 |
| SW | Postgraduate Programme Leader atLeedsMetropolitanUniversity | FSA and DEFRA Committees and working parties, including Cattle Movement and Food Borne DIsease, Consumer Representation for National Federation of Consumers | Secretary of the National Federation of Consumers, representative for the NFC on theInstituteofGrocery Distributors, and other FSA and Defra Committees | Leeds Metropolitan University | 0 |
| AW | Reader in Child Nutrition and Consultant in Neonatal Paediatrics,St George’sUniversityofLondon | Research Funding from Unicef | Chair of COMA’s Panel on Child & Maternal Nutrition and a member of COMA, member of the Food Standards Agency’s Expert Group on Vitamins and Minerals and cross-represented SACN on the Advisory Committee on Novel Foods and Processes between 2003 and 2007, chaired theNICECentre for Public Health Excellence Programme Development Group producing guidance on maternal and child nutrition in low-income families, represented SACN on the project board of the Diet and Nutrition Survey of Infants and Young Children | St Georges | 0 |
| HP | Professor of Nutritional Biochemistry, Head of Human Nutrition Unit, Deputy Head of Department of Oncology in the Faculty of Medicine, Dentistry and Health, University of Sheffield, | grants panels for Biotechnology and Biological Sciences Research Council (Agri-Food, Diet and Health Research Industry Club), Chair of grant panel for World Cancer Research Fund; a member of Expert Panel for the World Cancer Research Fund/Association, advisor to the Food Standards Agency for Biochemical Methodology for National Surveys of the Nutritional Status of the British Population |  | Sheffield | 0 |
| AW | Professor of Restorative Dentistry and Director of Research,SchoolofDental Sciences,NewcastleUniversity | Consultancy for GSK, Chlorhexidize Technology Inc. Reseach Funding for GSK |  | Newcastle | 3 |
| SLN | Reader in Nutrition at the University of Surrey | Consultancy for Kelloggs, Danone, Research Director and Shareholder of D3TEX LTD, Research Funding from Wassen International, Participated in ‘Health Hangout’ funded by Nestle, a web based discussion on vitamin D and bone health, specifically for health professionals. Small honorarium was received. |  | Surrey | 3 |
| JL | Professor of Metabolic Nutrition in the Department of Food and Nutritional Sciences and the Deputy Director of the Institute for Cardiovascular and Metabolic Research at theUniversityofReading | Serves on a number of research ethics committees. Research Funding from Unilever, Jordan, Nutricia, Sainsburys, Sugar Nutrition UK. Consultancy and Review writing for GSK, Research Funding from GSK |  | Reading | 3 |
| IY | Professor of Medicine and Director of the Centre for Public Health at Queen’s University Belfas | Associate Medical Director (Research and Development) and Consultant Chemical Pathologist at the Belfast Health and Social Care Trust, on the editorial boards of a number of leading international journals, Vice-Chair of the Scientific Division of the International Federation for Clinical Chemistry and Laboratory Medicine. Research Funding: Unilever, The Sugar Bureau |  | Queen's Belfast | 2 |
| HM | Deputy Director of Science and the Director of Academic Affairs at the Rowett Institute of Nutrition and Health,University of Aberdeen | Consultancy for EBRC (Chemical Industry, Germany) and SMBH (?Engineering, America?), Research funding from International Copper Association |  | Aberdeen | 3 |
| GF | Independent Public Health Nutritionist | Trustee of Institute of Food Research, Treasurer of Dementia Uk, Chair of NICE Group for Obese and Overweight Adults. Shareholder in Sainsburys, Consultancy work for Rodda's, Corporate Responsibility project work with Tesco. | Director of Consumer Choice and Dietary Health at the Food Standards Agency, served on several Government committees | - | 3 |
| MR | Food, Consumer Behaviour and Health Research Centre at theUniversityofSurrey | Expenses for expert advice: Choices International Foundation, Eurasante, European Commission DG Enterprise and Industry, Council for Responsible Nutrition - International. Research Funding from: Eurpean Commission DG Research and Innovation, European Food Information Council, MAPP - Centre for Research on Customer Relations in the Food Sector, Safefood, Food and Agriculture Organization | Institute of Food Research - Health Education Authority, National Centre for Health Outcomes Development at the University of Oxford, founding member nternational Society of Behavioral Nutrition and Physical Activity | Surrey | 2 |
| GP | Finance professional who has worked in both the accounting and fund management industries | Unknown - No Declaration |  |  | Unknown |

**Appendix 4 –** **Action on Sugar (AoS): Publicly Available Declarations of Interest**

| **Title & Initials** | **Employer (as listed on AoS webiste)** | **Other Employment/Interests** | **Significant Past Employment/Interests** | **Academic Institution** | **Rating (Based on publicly available CoI Declarations)** | |
| --- | --- | --- | --- | --- | --- | --- |
| GM | Professor of Cardiovascular Medicine at the Wolfson Institute, Queen Mary University of London | honorary consultant physician at St George’s Hospital, President of Consensus Action on Salt and Health (CASH) and World Action on Salt and Health (WASH), Chairman of Blood Pressure UK (BPUK) |  | Queen Mary University London | 0 | |
| AM | Cardiologist | Regularly writes for The Guardian |  |  | 0 | |
| RG | Co-director of the Human Nutrition Research Centre, Royal Victoria Infirmary, Newcastle |  | Formerly SACN | Newcastle | 0 | |
| AS | Emeritus Professor of Dental Public Health, School of Life and Medical Sciences, University College London |  |  | UCL | 0 | |
| DH | Chair, National Obesity Forum | Physician specialising in Obesity Medicine at the Centre for Obesity research at Luton & Dunstable Hospital. | Member of the Counterweight Scientific Advisory Board, acts as a consultant to Lighter Life, received honorariums for presentations and advisory board attendance from Sanofi-Aventis, Abbott, Roche and GlaxoSmithKline |  | 3 | |
| JC | Institute Director and Head of Centre for Cancer Prevention, Wolfson Institute of Preventive Medicine, Barts & The London School of Medicine |  | Oxford University and Columbia University, New York.  On AstraZeneca advisory board - institute receives support from AstraZeneca to conduct a prevention trial with anastrozole | Wolfson Institute | 3 | |
|
| JW | Professor of Endocrinology, Oxford University |  | Sub Dean, St Bart's Medical School | Oxford | 0 | |
| PS | Professor of Clinical Pharmacology & Therapeutics, Faculty of Medicine, National Heart & Lung Institute, Imperial College London | Co-Director of the International Centre for Circulatory Health, Honorary Consultant NHS Physician, member of CASH | General Medicine/ Cardiology Physician past-president of the British Hypertension Society and past president of European Council for Blood Pressure and Cardiovascular Research, served as consultant, received travel expenses from and payment for speaking at meetings for, and received research funding from Pfizer to cover administrative staffing and analytic costs of the biomarker analyses | Imperial | 3 | |
| PJ | Public Health policy Group and International Obesity Taskforce, London | President of IASO, member of CASH, member of International Obesity Taskforce | Senior lecturer at the London School of Hygiene and Tropical Medicine, Director of the Rowett Research Institute in Aberdeen | LSH&TM | 0 | |
| SC | Professor of Clinical Epidemiology, University of Liverpool | UK faculty Public Health, member of CASH, | NHS Physician | Liverpool | 0 | |
| NW | Professor of Environmental and Preventive Medicine, Wolfson Institute of Preventive Medicine, Barts & The London School of Medicine, London - |  | Holds patents for a combination pill for the prevention of cardiovascular disease | Wolfson Institite | 3 | |
| TL | Professor of Food Policy, University of London | Writes monthly column for 'The Grocer', member of CASH, member Food Climate Research Network | Consultant to WHO, consultant on food security to the Royal Institute of International Affairs, Commissioner on the UK Government's Sustainable Development Commission, council of Food Policy Advisors to Dept for Environment, Food & Rural Affairs | University of London | 0 | |
|
| RL | Professor of Pediatrics in the Division of Endocrinology at University of California, San Francisco | Author of 'Fat Chance' and other anti-food industry publications, Director of the Weight Assessment for Teen and Child Health (WATCH) Program, President of Institute for responsible nutrition | St. Jude Children’s Research Hospital in Memphis | UCSF | 0 | |
| YF | Assistant professor of Family Medicine, University of Ottawa | Medical Director, Bariatric Medical Institute, Author, Reality Coalition Canada | Family Medicine Chair, Canadian Obesity Network, Family Physician | Ottowa | 0 | |
| MR | Director of the British Heart Foundation Health Promotion Research Group, Nuffield Department of Population Health, University of Oxford | Chair of 'Sustain', trustee of UK health forum, member of FCRN | Head of Nutrition/Health MRC | Oxford | 0 | |
| JW | Retired Professor of Nutrition Policy, London Metropolitan University, London | Director of Food & Health Research at, an independent consultancy firm focused on nutrition that works primarily with public interest organisations, member of London Food Commission, National Food Alliance, Coronary Prevention Group, CASH, Joint Health Claims Initiative, Baby Drinks Campaign and Sustain, Advisory board of FAB research |  |  | 3 | |
| MK | Cambridge Food Control Ltd, Cambridge | NO DECLARATIONS FOUND |  |  | | Unknown – No declarations found |
| NR | Writer, journalist and NGO consultant, | Coordinates the International Obesity Forum | Former director of policy and public affairs at the International Obesity Task Force |  | 0 | |
| TF | Head Spokesperson, National Obesity Forum | Member of Child Growth Foundation |  |  | 0 | |
| PW | Professor of Cardiovascular Epidemiology, St George's, University of London | Served as a member of the British Heart Foundation Project Grant Committee, Pemberton Lecturer of the Society of Social Medicine | Member of the Sports Council for Northern Ireland , Member of the General Teaching Council for Northern Ireland, Development Director for Gymnastics Northern Ireland | St Georges | 0 | |
| RJ | Department of Medicine, University of Colorado Denver | Adjunct Professor of Medicine at the University of Florida, Author: The Sugar Fix |  | Colorado | 0 | |

**Appendix 5 –** **Heart of Mersey (HoM): Publicly Available CoI Declarations**

| **Title & Initials** | **Employer (as listed on HoM webiste)** | **Other Employment/Interests** | **Significant Past Employment/Interests** | **Academic Institution** | **Rating (Based on publicly available CoI Declarations)** |
| --- | --- | --- | --- | --- | --- |
| CB | Sefton PCT | University of Liverpool. | DPH of Stockport, senior lecturer at the University of Birmingham | Queen Mary University London | 0 |
| MB | Health Policy Resource Manager in the Joint Health Unit, based within Liverpool PCT |  | Finance Manager for Merseyside Health Action Zone, Liverpool First (Local Strategic Partnership) |  | 0 |
| SC | Professor of Clinical Epidemiology at University of Liverpool | UK faculty Public Health, CASH | NHS Physician, Glasgow University, has recently chaired/participated in a dozen national /international committees (including NICE, BHF, UK Faculty of Public Health and the European Society of Cardiology) | Newcastle | 0 |
| SD | consultant in Public Health for Liverpool PCT |  | Researcher for Manchester City Council, Health Promotion - Sefton | UCL | 0 |
| PD | Retired | WHO Advisor, member of the Committee of Management of the Benenden Healthcare Society | NHS Surgeon, Senior Medical Advisor DoH, developed the National Service Framework for CHD |  | 0 |
| LG | Director of Public Health for St Helens | lead for the children's agenda through the Health and Social Care Act transition on behalf of NHS Merseyside, leads the CHAMPS collaborative commissioning work stream and is a voting member of St Helens Clinical Commissioning Group Governing Body | NHS Nurse, NHS and Local Authority (Knowsley) | Wolfson Institite | 0 |
| JG | Manages communications team at 'Agent' |  | Common Purpose, Hilton Liverpool and Groundwork. | Oxford | unknown |
| PI | Senior Executive | Chair of HM Partnerships | 25 years experience in a complex, multi-national group working in commercial, strategic and change management role | Imperial | 0 |
| AJ | Honorary Past Chair' Alder Hey Childrens Hospital | Trustee for pioneering health and social care organisation PSS, Ronald McDonald House, Alder Hey | Non-Executive Director of Liverpool Family Health Services Authority, Chair of Liverpool Brook Advisory, Deputy Chair of Liverpool Health Authority and Chair of Liverpool Cardiothoracic Centre | LSH&TM | unknown |
| JP | PSS Head of Services for Health and Wellbeing |  | Neurosupport | Liverpool | Unknown |
| SS | consultant physician and cardiologist at the Royal Liverpool University Hospital |  | Clinical Director of medicine and the Clinical Sub-dean in the faculty of medicine at Liverpool University, Associate Medical Director for RLUH and Broadgreen hospitals, served on the Technology Appraisal Committee for the National Institute for Health and Clinical Excellence | Wolfson Institite | 0 |
| KW | Halton CCG | Writes monthly column for 'The Grocer', member of CASH, member Food Climate Research Network | NHS (Human Resources), Sefton PCT | University of London | 0 |

**Appendix 6**  **Sources for Declarations of Interest**

| **Group** | **Title & Initials** | **Source(s)** |
| --- | --- | --- |
| **ORG** | JA | http://www.instituteofhealthequity.org/about/the-team - http://www.london.gov.uk/moderngov/documents/g4304/Public%20reports%20pack%20Wednesday%2013-Jul-2011%2014.30%20Health%20and%20Public%20Services%20Committee.pdf?T=10 |
|  | LA | LinkedIn - http://www.medicalresearchfoundation.org.uk/about-us/board-of-trustees/louise-ansari/ - NO CoI DECLARATIONS FOUND |
|  | PA | LinkedIn - https://www.rcplondon.ac.uk/sites/default/files/action-on-obesity.pdf - http://www.phc.ox.ac.uk/team/researchers/paul-aveyard - http://www.sgul.ac.uk/research/researchers/u-z/michael-ussher/funding - http://www.addictionjournal.org/pages/ethical-policy - http://www.biomedcentral.com/1471-2458/14/620#sec4 - http://www.thecochranelibrary.com/details/editorial/1052523/Incentives-for-promoting-smoking-cessation-what-we-still-do-not-know.html - http://www.trialsjournal.com/content/15/1/296 - http://www.aso.org.uk/about-us/committee/ |
|  | JB | LinkedIn - NO CoI DECLARATIONS FOUND |
|  | SB | LinkedIn - http://hwb.birmingham.gov.uk/wp-content/uploads/2011/11/HWB-Minutes-4th-June-2013.pdf, http://hwb.birmingham.gov.uk/wp-content/uploads/2011/11/HWB-Minutes-4th-June-2013.pdf |
|  | CC | LinkedIn - NO CoI DECLARATIONS FOUND |
|  | SD | http://conversation.which.co.uk/author/sdavies/ - EFSA Annual Declaration of interests (ADoI) available at: https://ess.efsa.europa.eu/doi/doiweb/doisearch |
|  | CD | LinkedIn - http://www.bmj.com/content/344/bmj.e876.long - http://www.communityfoundation.org.uk/about/people/chris-drinkwater |
|  | KF | https://publichealthmatters.blog.gov.uk/author/kevin-fenton/ - http://www.bmj.com/content/349/bmj.g4785.long |
|  | NF | LinkedIn - https://www.rcplondon.ac.uk/sites/default/files/action-on-obesity.pdf - https://www.uclh.nhs.uk/OurServices/Consultants/Pages/ProfNickFiner.aspx |
|  | DH | http://en.wikipedia.org/wiki/David_Haslam_(GP) - http://www.ncbi.nlm.nih.gov/pmc/articles/PMC3553637/ |
|  | ZG | LinkedIn - NO CoI DECLARATIONS FOUND |
|  | AH | http://medhealth.leeds.ac.uk/profile/600/527/professor_andrew_hill - http://www.nature.com/ijo/journal/v37/n3/full/ijo201274a.html |
|  | PI | http://www.nice.org.uk/guidance/ph41/resources/walking-and-cycling-pdg-12 |
|  | SJ | http://www.phc.ox.ac.uk/team/researchers/susan-jebb - https://www.rcplondon.ac.uk/sites/default/files/action-on-obesity.pdf - often declares no interests in academic papers - published in 2010 in the American Journal of Clinical Nutrition: SAJ [Susan Jebb] is a member of Scientific Advisory Boards for Coca-Cola, Heinz, PepsiCo, Nestlé and Kellogg’s. |
|  | ML | http://www.carnegieuktrust.org.uk/who-we-are/staff---trustees - NO CoI DECLARATIONS FOUND |
|  | PL | http://www.ukhealthforum.org.uk/who-we-are/our-people/ - http://www.nice.org.uk/guidance/gid-phg57/resources/workplace-policy-and-management-practices-to-improve-the-health-of-employees-final-minutes-of-phac-1-meeting2 |
|  | IM | LinkedIn - http://www.sacn.gov.uk/about_us/members_biographies.html - http://www.sacn.gov.uk/pdfs/sacn_annual_report_2012.pdf - https://www.gov.uk/government/uploads/system/uploads/attachment_data/file/273719/451_FOI_Annex_1.pdf - |
|  | MM | http://www.science.ulster.ac.uk/sesri/profiles/m.murphy.php - http://www.sciencedirect.com/science/article/pii/S193317111300171X |
|  | AO | http://www.brc.org.uk/brc_directors.asp - NO CoI DECLARATIONS FOUND |
|  | SP | NO INFORMATION FOUND |
|  | DR | Inferred from: http://www.nice.org.uk/guidance/ta282/chapter/9-Appraisal-Committee-members-guideline-representatives-and-NICE-project-team |
|  | AR | LinkedIn - https://democratic.trafford.gov.uk/ieListDocuments.aspx?CId=260&MId=819&Ver=4 - http://www.traffordccg.nhs.uk/wp-content/uploads/2014/05/AI-3.2-Register-of-interests-2.pdf |
|  | MS | http://nnet-server.com/server/common/eacouncil141.htm - http://ealing.cmis.uk.com/Ealing/Document.ashx?czJKcaeAi5tUFL1DTL2UE4zNRBcoShgo=8HVqwhptPUJL93%2F5LYz5IFBPjKR2LL9xGBVhqTZcOMTDMbKSPwwyrQ%3D%3D&rUzwRPf%2BZ3zd4E7Ikn8Lyw%3D%3D=pwRE6AGJFLDNlh225F5QMaQWCtPHwdhUfCZ%2FLUQzgA2uL5jNRG4jdQ%3D%3D&mCTIbCubSFfXsDGW9IXnlg%3D%3D=hFflUdN3100%3D&kCx1AnS9%2FpWZQ40DXFvdEw%3D%3D=hFflUdN3100%3D&uJovDxwdjMPoYv%2BAJvYtyA%3D%3D=ctNJFf55vVA%3D&FgPlIEJYlotS%2BYGoBi5olA%3D%3D=NHdURQburHA%3D&d9Qjj0ag1Pd993jsyOJqFvmyB7X0CSQK=ctNJFf55vVA%3D&WGewmoAfeNR9xqBux0r1Q8Za60lavYmz=ctNJFf55vVA%3D&WGewmoAfeNQ16B2MHuCpMRKZMwaG1PaO=ctNJFf55vVA%3D |
|  | TS | https://www.fmlm.ac.uk/terence-stephenson - http://www.mhra.gov.uk/home/groups/dir/documents/websiteresources/con432919.pdf - http://www.theguardian.com/society/2012/jul/24/terence-stephenson-doctors-nhs |
|  | JV | http://www.england.nhs.uk/about/whos-who/ncd/#obe - http://www.plosone.org/article/info%3Adoi%2F10.1371%2Fjournal.pone.0022142 |
|  | JW | https://iris.ucl.ac.uk/iris/browse/profile?upi=FJWAR41 - http://en.wikipedia.org/wiki/Jane_Wardle - http://link.springer.com/article/10.1007/s10897-013-9628-9/fulltext.html |
|  | CG | https://www.rcm.org.uk/college/about/media-centre/press-releases/doncaster-midwives-win-top-uk-award-tackling-obesity-in - http://shura.shu.ac.uk/4005/1/1471-2393-11-69.pdf |
|  |  |  |
| **SACN** | AP | http://www.sacn.gov.uk/about_us/members_biographies.html - http://www.sacn.gov.uk/pdfs/sacn_annual_report_2012.pdf |
|  | PA | http://www.sacn.gov.uk/about_us/members_biographies.html - http://www.sacn.gov.uk/pdfs/sacn_annual_report_2012.pdf |
|  | PH | http://www.sacn.gov.uk/about_us/members_biographies.html - http://www.sacn.gov.uk/pdfs/sacn_annual_report_2012.pdf |
|  | TK | http://www.sacn.gov.uk/about_us/members_biographies.html - http://www.sacn.gov.uk/pdfs/sacn_annual_report_2012.pdf |
|  | DM | http://www.sacn.gov.uk/about_us/members_biographies.html - http://www.sacn.gov.uk/pdfs/sacn_annual_report_2012.pdf |
|  | IM | http://www.sacn.gov.uk/about_us/members_biographies.html - http://www.sacn.gov.uk/pdfs/sacn_annual_report_2012.pdf |
|  | SW | http://www.sacn.gov.uk/about_us/members_biographies.html - http://www.sacn.gov.uk/pdfs/sacn_annual_report_2012.pdf |
|  | AW | http://www.sacn.gov.uk/about_us/members_biographies.html - http://www.sacn.gov.uk/pdfs/sacn_annual_report_2012.pdf |
|  | HP | http://www.sacn.gov.uk/about_us/members_biographies.html - http://www.sacn.gov.uk/pdfs/sacn_annual_report_2012.pdf |
|  | AW | http://www.sacn.gov.uk/about_us/members_biographies.html - http://www.sacn.gov.uk/pdfs/sacn_annual_report_2012.pdf |
|  | SLN | http://www.sacn.gov.uk/about_us/members_biographies.html - http://www.sacn.gov.uk/pdfs/sacn_annual_report_2012.pdf |
|  | JL | http://www.sacn.gov.uk/about_us/members_biographies.html - http://www.sacn.gov.uk/pdfs/sacn_annual_report_2012.pdf |
|  | IY | http://www.sacn.gov.uk/about_us/members_biographies.html - http://www.sacn.gov.uk/pdfs/sacn_annual_report_2012.pdf |
|  | HM | http://www.sacn.gov.uk/about_us/members_biographies.html - http://www.sacn.gov.uk/pdfs/sacn_annual_report_2012.pdf |
|  | GF | http://www.sacn.gov.uk/about_us/members_biographies.html - http://www.sacn.gov.uk/pdfs/sacn_annual_report_2012.pdf |
|  | MR | http://www.sacn.gov.uk/about_us/members_biographies.html - http://www.sacn.gov.uk/pdfs/sacn_annual_report_2012.pdf |
|  | GP | NO INFORMATION FOUND |
|  |  |  |
| **AoS** | GM | http://www.euro.who.int/en/media-centre/events/events/2013/07/vienna-conference-on-nutrition-and-noncommunicable-diseases/biographies/professor-graham-macgregor - http://www.sciencedirect.com/science/article/pii/S014067361362468X , |
|  | AM | http://www.theguardian.com/profile/aseem-malhotra - http://press.psprings.co.uk/bmj/may/Malhotra.pdf |
|  | ARG | http://www.sacn.gov.uk/pdfs/sacn_04_03min.pdf - http://www.ama.ba/index.php/ama/article/viewFile/183/pdf_17 |
|  | AS | <http://www.plosone.org/article/info%3Adoi%2F10.1371%2Fjournal.pone.0104808> |
|  | DH | http://en.wikipedia.org/wiki/David_Haslam_(GP) , http://www.ncbi.nlm.nih.gov/pmc/articles/PMC3553637/ |
|  | JC | http://www.ncbi.nlm.nih.gov/pmc/articles/PMC3017674/ - http://www.ncbi.nlm.nih.gov/pmc/articles/PMC3005729/ |
|  | JW | http://www.ocdem.ox.ac.uk/grant-holders/researcher/john-wass - https://www.rcplondon.ac.uk/sites/default/files/action-on-obesity.pdf |
|  | PS | http://www.imperial.ac.uk/AP/faces/pages/read/Home.jsp?person=p.sever&_adf.ctrl-state=194hctkq7v_3&_afrRedirect=1134217940170235 - http://hyper.ahajournals.org/content/63/3/507.full |
|  | PJ | http://www.debretts.com/people-of-today/profile/926/(William)-Philip-Trehearne-JAMES - http://www.euro.who.int/en/media-centre/events/events/2013/07/vienna-conference-on-nutrition-and-noncommunicable-diseases/biographies/dr-philip-james - https://www.rcplondon.ac.uk/sites/default/files/action-on-obesity.pdf - http://www.actiononsalt.org.uk/about/CASH%20Members/index.html |
|  | SC | http://www.liv.ac.uk/psychology-health-and-society/staff/simon-capewell/ - http://www.sciencedirect.com/science/article/pii/S0140673613626057 - http://www.actiononsalt.org.uk/about/CASH%20Members/index.html |
|  | NW | <http://www.plosone.org/article/info:doi/10.1371/journal.pone.0018742> |
|  | TL | http://www.city.ac.uk/people/academics/timothy-lang - https://www.rcplondon.ac.uk/sites/default/files/action-on-obesity.pdf - http://www.actiononsalt.org.uk/about/CASH%20Members/index.html - http://www.fcrn.org.uk/about/advisory-board |
|  | RL | http://profiles.ucsf.edu/robert.lustig - http://www.plosone.org/article/info%3Adoi%2F10.1371%2Fjournal.pone.0057873 - http://www.responsiblefoods.org/ |
|  | YF | LinkedIn - http://onlinelibrary.wiley.com/doi/10.1111/obr.12128/full |
|  | MR | http://onlinelibrary.wiley.com/doi/10.1111/obr.12142/full - http://www.dph.ox.ac.uk/bhfhprg/members/academic/mike-rayner , http://www.fcrn.org.uk/about/advisory-board |
|  | JW | http://www.bmj.com/content/344/bmj.e2931/rr/585808 - http://www.bmj.com.ezproxy.liv.ac.uk/content/348/bmj.g3204.long - http://www.fabresearch.org/viewItem.php?id=7431 |
|  | MK | NO DECLARATION FOUND |
|  | NR | http://www.theguardian.com/profile/nevillerigby - http://www.actiononsalt.org.uk/actiononsugar/index.html - http://www.ncbi.nlm.nih.gov/pmc/articles/PMC3820069/ - http://www.researchgate.net/profile/Neville_Rigby |
|  | TF | http://www.nationalobesityforum.org.uk/index.php/contact_the_nof.html - http://www.nature.com/ijo/journal/v34/n4/full/ijo2009237a.html |
|  | PW | http://www.sgul.ac.uk/research/researchers/u-z/peter-whincup - http://www.ncbi.nlm.nih.gov/pmc/articles/PMC3661837/ |
|  | RJ | http://www.foodaddictionsummit.org/presenters-johnson.htm - http://www.foodaddictionsummit.org/docs/johnson-347ajcn%20review.pdf |
|  |  |  |
| **HoM** | CB | http://www.heartofmersey.org.uk/trustees.asp - https://www.rcplondon.ac.uk/sites/default/files/action-on-obesity.pdf |
|  | MB | http://www.heartofmersey.org.uk/trustees.asp - http://www.heartofmersey.org.uk/uploadedfiles/documents/Trustees_minutes_March_2010.pdf |
|  | SC | http://www.heartofmersey.org.uk/trustees.asp - http://www.sciencedirect.com/science/article/pii/S0140673613626057 |
|  | SD | http://www.heartofmersey.org.uk/trustees.asp - http://www.liverpoolccg.nhs.uk/Library/About_us/Board/2014/WebsitePackLCCGGoverningBody8July2014PapersPack.pdf |
|  | PD | http://www.heartofmersey.org.uk/trustees.asp - http://www.heartofmersey.org.uk/uploadedfiles/documents/Trustees_minutes_September_2010.pdf |
|  | LZ | http://www.heartofmersey.org.uk/trustees.asp - http://www.sthelensccg.nhs.uk/Library/public_info/Governing_Body_papers/2014/Governing_Body_Meeting_17.7.14_Part_I.pdf |
|  | JG | http://www.heartofmersey.org.uk/trustees.asp - NO DECLARATIONS FOUND |
|  | PI | http://www.heartofmersey.org.uk/trustees.asp - http://www.heartofmersey.org.uk/uploadedfiles/documents/Trustees_minutes_March_2010.pdf |
|  | AJ | http://www.heartofmersey.org.uk/trustees.asp - NO DECLARATIONS FOUND |
|  | JP | http://www.heartofmersey.org.uk/trustees.asp - NO DECLARATIONS FOUND |
|  | SS | http://www.heartofmersey.org.uk/trustees.asp - http://www.heartofmersey.org.uk/uploadedfiles/documents/Trustees_minutes_March_2011.pdf |
|  | KW | http://www.heartofmersey.org.uk/trustees.asp - http://www.haltonccg.nhs.uk/Library/public_information/January_2014/Public%20Papers%20for%20020114%20Governing%20Body%20Meeting.pdf |

**Appendix 7 Identifying publicly accessible CoI declarations**

| **Systematic Google searches** |
| --- |
| *‘NAME’ + ‘Conflict+of+interest’* |
| *‘NAME’ + ‘Competing+interest’* |
| *‘NAME’ + ‘Declaration+of+interest’* |
| *‘NAME’ + ‘Conflict+of+interest’ + ‘meeting’* |
| *‘NAME’ + ‘Competing+interest’ + ‘meeting’* |
| *‘NAME’ + ‘Declaration+of+interest’ + ‘meeting’* |

| **Systematic Google Scholar searches** |
| --- |
| ‘AUTHOR NAME’ + food policy (all of the words anywhere in the article) + year published after 2009 |
| ‘AUTHOR NAME’ + nutrition (anywhere in the article) + year published after 2009 |

Relevant web pages/documents resulting from these searches were identified and analysed by AN.

**Appendix 8 Rapid Review: Managing Conflicts of Interest, Potential Strategies**

We identified 115 potentially relevant papers through the SCOPUS database review. The majority concerned conflict of interest (CoI) in pharmaceutical, alcohol and tobacco industries rather than food or agriculture. These CoI affected policy makers plus a wide range of professions, including clinicians, researchers, guideline developers, and journal editors.

We initially identified 28 papers which were subject to the inclusion criteria for the scoping review (Table 1). Table 2 and 3 provide the characteristics and main results of the included studies respectively. Of the 28 papers identified, 26 papers proposed approaches for managing CoI. There is currently no validated classification system for different approaches to managing CoI. We therefore created a novel typology ranking and classifying each paper as ‘deny’, (ignore or conceal), ‘describe’ (document then dismiss) or ‘diminish’ (actively identify, minimise or prevent) (Table 4).

Overall, 65% of the 26 studies recommended introducing some form of “diminish” policy. The most popular recommendation was to improve transparency (81%). Only one study recommended purely “diminish” changes to manage CoI (Table 5).

Most authors recommended rigorous policies for identifying and managing CoI and restricting interactions with industry. However, merely having a policy on CoI may not be sufficient to prevent their negative effects. The stringency of current policies varied greatly between institutions and adherence was often reported to be poor^1^.

1. Warner TD, Gluck JP. What do we really know about conflicts of interest in biomedical research? *Psychopharmacol*. 2003;171(1):36-46.

**Table 1 - Literature Review Search Terms and Inclusion Criteria**

| **SCOPUS search terms** | #1 Industr* OR Commerc* |
| --- | --- |
|  | #2 ‘Conflict of interest OR competing interest’ |
|  | #3 ‘Policy OR guid*’ |
|  | #4 ‘Bias OR influen* NOT influenz*’ |
| **SCOPUS search terms: #1 AND #2 AND #3 AND #4:** |  |
|  | 1. Industr* OR Commerc* |
|  | 2. Conflict of interest OR competing interest |
|  | 3. Policy OR guid* |
|  | 4. Bias OR influen* NOT influenz* |
|  |  |
|  | (all within Title/Abstract/keywords) |

| **Inclusion Criteria** | Article Type: Review |
| --- | --- |
|  | Source Type: Journal |
|  | English Language |
|  | Relating to food, alcohol, pharmaceutical or tobacco industry |
|  | Focus on policy making preferred |
|  | Quantitative data preferred |
|  | Systematic reviews and primary research |

**Table 2 –Characteristics of Included Studies**

| **Study No.** | **Title** | **Lead Author** | **Year** | **Journal** |
| --- | --- | --- | --- | --- |
| 1 | Interactions between Non-Physician Clinicians and Industry: A Systematic Review | Grundy, Q. | 2013 | PLoS Medicine |
| 2 | How experts are chosen to inform public policy: Can the process be improved? | Rowe, S. | 2013 | Health Policy |
| 3 | Addiction industry studies: Understanding how proconsumption influences block effective interventions | Adams, P.J. | 2013 | American Journal of Public Health |
| 4 | Systematic analysis of hydroxyethyl starch (HES) reviews: Proliferation of low-quality reviews overwhelms the results of well-performed meta-analyses | Hartog, C.S. | 2012 | Intensive Care Medicine |
| 5 | Perceptions of conflict of interest disclosures among peer reviewers | Lippert, S. | 2011 | PLoS 1 |
| 6 | Enhancing ties between academia and industry to improve health | Johnston, S.C. | 2011 | Nature Medicine |
| 7 | Saving U.S. dietary advice from conflicts of interest | Herman, J. | 2010 | Food and Drug Law Journal |
| 8 | A critical review of the American pain society clinical practice guidelines for interventional techniques: Part 1. Diagnostic interventions | Manchikanti, L. | 2010 | Pain Physician |
| 9 | The impact of disclosing financial ties in research and clinical care: A systematic review | Licurse, A. | 2010 | Archives of Internal Medicine |
| 10 | Partial progress: Governing the pharmaceutical industry and the NHS, 1948-2008 | Abraham, J. | 2009 | Journal of Health Politics, Policy and Law |
| 11 | Addressing conflict in strategic literature reviews: Disclosure is not enough | Michaels, D. | 2009 | Journal of Epidemiology and Community Health |
| 12 | Professional medical associations and their relationships with industry: a proposal for controlling conflict of interest | Rothman, D.J. | 2009 | JAMA |
| 13 | Are self-regulation and declaration of conflict of interest still the benchmark for relationships between physicians and industry? | Haines, I.E. | 2008 | Medical Journal of Australia |
| 14 | Evaluating solutions to sponsorship bias | Doucet, M. | 2008 | Journal of Medical Ethics |
| 15 | A turning point for conflicts of interest: The controversy over the National Academy of Sciences' first conflicts of interest disclosure policy | Parascandola, M. | 2007 | Journal of Clinical Oncology |
| 16 | Industry influence on occupational and environmental public health | Huff, J. | 2007 | International Journal of Occupational and Environment Health |
| 17 | Identifying carcinogens: The tobacco industry and regulatory politics in the United States | Cook, D.M. | 2006 | International Journal of Health Services |
| 18 | Advertising and disclosure of funding on patient organisation websites: A cross-sectional survey | Ball, D.E. | 2006 | BMC Public Health |
| 19 | Lifting the veil of secrecy from industry funding of nonprofit health organizations | Jacobson, M.F. | 2005 | International Journal of Occupational and Environment Health |
| 20 | What do we really know about conflicts of interest in biomedical research? | Warner, T.D. | 2003 | Psychopharmacology |
| 21 | Financial conflict-of-interest policies in clinical research: Issues for clinical investigators | Boyd, E.A. | 2003 | Academic Medicine |
| 22 | Science for hire: A tobacco industry strategy to influence public opinion secondhand smoke | Muggli, M.E. | 2003 | Nicotine and Tobacco Research |
| 23 | Scope and impact of financial conflicts of interest in biomedical research: A systematic review | Bekelman, J.E. | 2003 | JAMA |
| 24 | Is the fox guarding the henhouse? Who makes the rules in american nutrition policy? | Schaffer, E.J. | 2002 | Food and Drug Law Journal |
| 25 | The pharmaceutical industry as a political player | Abraham, J. | 2002 | Lancet |
| 26 | Cancer-related health policy: beyond the smoke and mirrors. | Parsons, S.K. | 2002 | Seminars in Oncology Nursing |
| 27 | Research funding, conflicts of interest, and the "meta-methodology" of public relations | Rampton, S. | 2002 | Public Health Reports |
| 28 | Food lobbies, the food pyramid, and U.S. nutrition policy | Nestle, M. | 1993 | International Journal of Health Services |

**Table 3 –Main Results**

| **No.** | **Subject Focus** | **Industry Focus** | **Prevalence of CoI** | **Perception of CoI** | **Impact of CoI** | **Authors comment** |
| --- | --- | --- | --- | --- | --- | --- |
| 1 | Clinicians (non-physicians) | Pharma | Clinicians across disciplines met with pharmaceutical representatives regularly and relied on them for practice information and acted as distributors. | Ethical use of industry resources, and felt they could detect "promotion" while benefiting from industry "information." |  | Industry interactions normalised over many disciplines |
| 2 | Policy makers | Food/ Nutrition |  |  |  |  |
| 3 | Policy Makers | Alcohol/ Tobacco | Profits from these substances are deployed in ways that block or divert resources from interventions known to be effective |  |  |  |
| 4 | Researchers | Pharma | Favourable reviews increased from two to eight per year | Fourteen authors published the majority (70/124) of positive reviews, and ten of these 14 had or have since developed a pCOI with various manufacturers of HES | Market tripled from 20 to 60 % -The rates of recommending against HES use are 83 % (10/12) in meta-analyses and 20 % (31/153) in reviews without meta-analysis | Low-quality HES reviews reached different conclusions than high-quality meta-analyses from independent entities - The majority of these low-quality positive HES reviews were written by a small group of authors, most of whom had or have since established ties to industry |
| 5 | Peer Reviewers | Perception of CoI |  | 50% would not affect chance of publication - Those with CoI less likely to consider CoI problematic |  |  |
| 6 | Researchers | Pharma/ Devices | - | Concerns about conflicts of interest have driven a wedge between academia and the pharmaceutical and devices industries | - | partnerships between industry and academia are essential |
| 7 | Policy makers | Food/ Agriculture | Very poor transparency | High suspicion as guidelines differ from scientific consensus | Guidelines are ineffective at improving health | Integrity has been compromised |
| 8 | Guideline Developers | Pharma/ Devices | The reassessment illustrates that Chou et al have utilized multiple studies inappropriately and have excluded appropriate studies. Also, Chou et al failed to eliminate their bias in their study evaluations. |  | Patient Care has been sacrificed for profit | The reassessment, using appropriate methodology and including high quality studies, shows evidence that differs from published APS guidelines. |
| 9 | Patients, Clinicians, Research Participants | Generic |  | Patients believed FTs decreased the quality and increased the cost of care - perceptions of journal article quality decreased after disclosure of FTs - Patients were more likely to view personal gifts to physicians as unacceptable, compared with professional gifts - most patients and research participants believed FTs should be disclosed - one-quarter of participants reported less willingness to participate in after disclosure of FTs | Disclosure: Patients believe that FTs influence professional behaviour and should be disclosed. Patients, physicians, and research participants believe FTs decrease the quality of research evidence, and, for some, knowledge of FTs would affect willingness to participate in research |  |
| 10 | NHS | Pharma | Enormous and unrivalled influence afforded to the pharmaceutical industry in policy developments |  | Progress has been slow, restricted, and vulnerable enormous and unrivalled influence afforded to the pharmaceutical industry in policy developments |  |
| 11 | Journals/ Editors | Generic | Many institutions are introducing or updating disclosure of CoI policies | Peer review is generally unable to detect fraud, and is not a guarantee of either accuracy or objectivity |  | The credibility given strategic reviews by publication in peer-reviewed journals is undeserved, and potentially hazardous to public health. |
| 12 | Professional Medical Associations | Pharma/ Devices | Many PMAs receive extensive funding from pharmaceutical and device companies | Current PMA policies, however, are not uniform and often lack stringency | Their conferences, continuing medical education courses, practice guidelines, definitions of ethical norms, and public advocacy positions carry great weight with physicians and the public | Nevertheless, these changes are in the best interest of the PMAs, the profession, their members, and the larger society |
| 13 | Clinicians, Research | Pharma |  |  | Positive association between pharmaceutical industry sponsorship and reporting of positive outcomes, manipulation of clinical trials, and hiding of "preliminary data sets" - Influential literature reviews and treatment guidelines have been associated with widespread declarations of conflict of interest | Transparency alone may not be sufficient to erase the doubts created when authors of clinical practice guidelines or editorials declare potential conflicts of interest |
| 14 | Researchers | Pharma |  |  | Industry sponsorship biases published scientific research in favour of the sponsors, | Financial disclosure, reporting standards and trial registries either fail to address the mechanisms or else only inadequately address those mechanisms |
| 15 | Researchers | Generic | POLICY: committee members to disclose any "potential sources of bias" that "others might deem prejudicial. | Scientists universally opposed the policy | All experienced and knowledgeable experts were inherently conflicted, others were offended at the suggestion that any expert could be biased' |  |
| 16 | Researchers/Clinicians | Generic | Increasing control by industry interests |  | Government has failed to support independent, public health-oriented practitioners and their organizations, instead joining many corporate endeavours to discourage efforts to protect the health of workers and the community | More overt influence nowadays |
| 17 | Policy Makers | Tobacco | Vigorously contested a specialized question |  | Despite industry efforts to challenge both the substance of the report and the agency procedures, environmental tobacco smoke was declared by the agency in 2000 to be a known human carcinogen | In this case, tobacco industry regulation contradicts academic expectations of business regulatory victories. However, the tobacco industry's participation in the regulatory process influenced the process in favor of all regulated industry. |
| 18 | Professional Medical Associations | Pharma | Disclosure of donors varied substantially. Corporate donations were itemised in 7/37 reports - none gave enough information to show the proportion of funding from Pharma. 45% declared Pharma funding but annual reports named more than the websites (median 6 vs. 1). One third of websites showed one or more company logos and/or had links to Pharma websites. Pharma introductions were present on 10% of websites, some of them mentioning specific products. Two patient organisations had obvious close ties to Pharma. |  |  | Patient organisation websites do not provide enough information for visitors to assess whether a conflict of interest with Pharma exists |
| 19 | Professional Medical Associations, non-profit health groups | Generic | In some cases, industry appears either to influence an organization's positions or to limit an organization's freedom |  |  | Non-profit organizations need to consider the potential influence on their independence if they accept funding |
| 20 | Researchers | Generic | At least 30% - the rate of disclosure of conflicts of interest is as low as 2% (in specific situations - not tested for accuracy)` | Some evidence exists to indicate that researchers with conflicting interests may indeed offer different professional opinions and judgments than those for whom such conflicts do not exist |  |  |
| 21 | Clinical Investigators | Generic | As industry sponsorship of clinical research grows, investigators' personal financial relationships with those sponsors are under increasing scrutiny | Fewer than half of the interviewed investigators could accurately describe their campus' conflict-of-interest policy. Many investigators felt that professional societies, the public, and individual investigators were appropriate monitors of conflicts of interest. Many investigators recognized the general risks associated with conflicts of interest, but felt that they personally were not at risk |  |  |
| 22 | Researchers/Consultants | Tobacco | Created an international scientific consultants program to influence public opinion | A "product" to protect the industry from international threats of smoking restrictions | Promote a scientific backdrop supporting the industry's position on ETS that differed from regulatory agencies and published scientific research |  |
| 23 | Researchers | Generic | 2/3 academic institutions and 1/4 investigators have fCoI | The approach to managing financial conflicts varied substantially across academic institutions and peer-reviewed journals | Odds ratio: 3.60 (Chances of industry positive result) - Industry sponsorship was also associated with restrictions on publication and data sharing |  |
| 24 | Policy Makers | Food/ Agriculture | Increasing numbers of members with commercial CoI |  | Nutritional interests of the American public may not be well served by government agencies charged with both encouraging healthful dietary habits and promoting American agricultural products | The failure to question this alliance's continuing validity in an era of plenty has rendered policy makers unduly susceptible to the influence of food industry lobbyists |
| 25 | Government | Pharma |  | The extent of industry influence over drug regulation, at the Expense of other interested parties, suggests that the current system could be more robust |  |  |
| 26 | Guideline Developers | Pharma | Special interest groups of all sizes and causes interface with the legislative process to influence outcome at every level of policy development, implementation, and evaluation |  | Guidelines are formulated and actualized being influenced by competing interests, needs, and resources, which is an inherently political process. |  |
| 27 | PR firm tactics | Generic |  | Transparent disclosure does not prevent CoI harms |  |  |
| 28 | Policy Makers | Food/ Agriculture |  |  | Since 1977, under pressure from meat producers, federal dietary advice has evolved from "decrease consumption of meat" to "have two or three (daily) servings. | Conflict between federal protection of the rights of food lobbyists to act in their own self-interest and federal responsibility to promote the nutritional health of the public |

**Table 4 – Proposed Typology of Management of Conflicts of Interest**

| **Standpoint** | **Suggested Management** | **Number of Studies Recommending Suggested Management** | **%** |
| --- | --- | --- | --- |
| **Deny** | Do Nothing | 4 | 15 |
|  | Increase Self-Regulation and/or Professional Standards | 11 | 42 |
| **Describe** | Education on CoI | 5 | 19 |
|  | Improve Transparency | 21 | 81 |
|  | Introduce Central Repository for industry Funding | 3 | 12 |
|  | Standard CoI Policy Across Multiple Centres/Disciplines | 4 | 15 |
| **Diminish** | Prevent/Limit Industry Interaction | 15 | 58 |
|  | Independent Non-Conflicted Group to Review CoI | 11 | 42 |
|  | Sanctions for Those Who Breach Policy | 2 | 8 |

**Table 5 –** **Managing Conflicts of Interest, Characteristics of Included Studies and Classification According to Proposed Typology**

| **Study No.** | **Title** | **Lead Author** | **Year** | **Journal** | **Standpoint (Using Proposed Typology)** |
| --- | --- | --- | --- | --- | --- |
| 1 | Conflicts of interest in psychiatry: Strategies to cultivate literacy in daily practice | Rumiko Shimazawa | 2014 | Psychiatry and Clinical Neuroscience | Diminish |
| 2 | How experts are chosen to inform public policy: Can the process be improved? | Rowe, S. | 2013 | Health Policy | Describe |
| 3 | Conflict of interest and professional medical associations: The North American Spine Society experience | Schofferman, J.A. | 2013 | Spine Journal | Diminish |
| 4 | Conflict of interest reporting in otolaryngology clinical practice guidelines | Sun, G.H. | 2013 | Otolaryngology - Head and Neck Surgery (United States) | Describe |
| 5 | Disclosures of conflicts of interest in psychiatric review articles | Kopelman, A.M. | 2013 | Journal of Nervous and Mental Disease | Diminish |
| 6 | A humble task: Restoring virtue in an age of conflicted interests | Dubois, J.M. | 2013 | Academic Medicine | Describe |
| 7 | Perceptions of conflict of interest disclosures among peer reviewers | Lippert, S. | 2011 | PLoS 1 | Describe |
| 8 | Disclosure of industry relationships by anesthesiologists: Is the conflict of interest resolved? | Kofke, W.A. | 2010 | Current Opinion in Anaesthesiology | Describe |
| 9 | From quid pro quo to quid pro bono: Reshaping the influence of industry on health care epidemiologists | Kirkland, K.B. | 2010 | Clinical Infectious Diseases | Diminish |
| 10 | Can academic departments maintain industry relationships while promoting physician professionalism? | Dubovsky, S.L., | 2010 | Academic Medicine | Diminish |
| 11 | Limiting the influence of pharmaceutical industry gifts on physicians: Self-regulation or government intervention? | Grande, D. | 2010 | Journal of General Internal Medicine | Deny |
| 12 | Financial and intellectual conflicts of interest: Confusion and clarity | Bion, J. | 2009 | Current Opinion in Critical Care | Describe |
| 13 | Funding food science and nutrition research: Financial conflicts and scientific integrity | Rowe, S. | 2009 | Nutrition reviews | Diminish |
| 14 | Professional medical associations and their relationships with industry | Rothman, D.J. | 2009 | JAMA | Diminish |
| 15 | A common standard for conflict of interest disclosure in addiction journals | Goozner, M. | 2009 | Addiction | Describe |
| 16 | Are self-regulation and declaration of conflict of interest still the benchmark for relationships between physicians and industry? | Haines, I.E. | 2008 | Medical Journal of Australia | Diminish |
| 17 | Evaluating solutions to sponsorship bias | Doucet, M. | 2008 | Journal of Medical Ethics | Diminish |
| 18 | Collaborations between academic psychiatry and the pharmaceutical industry: A perspective from industry | Tohen, M. | 2007 | Epidemiologia e Psichiatria Sociale | Deny |
| 19 | Counterpoint: Physician-industry relationships can be ethically established, and conflicts of interest can be ethically managed | White, A.P. | 2007 | Spine | Describe |
| 20 | Potential for conflict of interest in the evaluation of suspected adverse drug reactions: Use of cerivastatin and risk of rhabdomyolysis | Psaty, B.M. | 2004 | JAMA | Diminish |
| 21 | Using the literature in developing McGill's guidelines for interactions between residents and the pharmaceutical industry | Wazana, A. | 2004 | Academic Medicine | Diminish |
| 22 | What do we really know about conflicts of interest in biomedical research? | Warner, T.D. | 2003 | Psychopharmacology | Diminish |
| 23 | Financial conflict of interest in medical research: Overview and analysis of institutional controls | Henderson, J.A. | 2003 | Food and Drug Law Journal | Describe |
| 24 | The pharmaceutical industry as a political player | Abraham, J. | 2002 | Lancet | Diminish |
| 25 | Research funding, conflicts of interest, and the "meta-methodology" of public relations | Rampton, S. | 2002 | Public Health Reports | Describe |
| 26 | Ethical issues concerning the relationships between medical practitioners and the pharmaceutical industry | Komesaroff, P.A. | 2002 | Medical Journal of Australia | Diminish |

**Appendix 9 Analysis of SACN meeting minutes**

Minutes from SACN archives (<http://webarchive.nationalarchives.gov.uk/20140507012718/http://www.sacn.gov.uk/meetings/committee/main_sacn_meetings/index.html>) analysed. Mentions of CoI summarised in table along with any evidence of action taken that was noted.

| **Meeting No.** | **Date** | **Mention of CoI** | **Evidence of action taken** |
| --- | --- | --- | --- |
| 27 | 10/06/2009 | None | n/a |
| 28 | 01/10/2009 | None | n/a |
| 29 | 02/10/2009 | Concern over legal implications of contrasting conclusions between SACN and ESFA reports. Some members noted to have sat on EFSA committees | FSA observer explains role of EFSA and offers to present a paper on the subject at a future date. EFSA should also provide updates on its work for consideration at SACN meetings. Both points are fulfilled next meeting |
| 30 | 24/02/2010 | Members made no new declarations of interest.  Possibility of increasing openness of discussions to aid public discussion considered | Group votes for maintaining status quo, ‘horizon scanning’ and items that are pre-consultation or include confidential or unpublished data discussed in closed session.  Members agreed that post-consultation reports, generally, should be finalised and agreed by the Committee in open session in the future |
| 31 | 07/06/2010 | No updates to declarations of interest | None |
| 32 | 15/10/2010 | No updates to declarations of interest.  New chair Ann Prentice advised that if there were any agenda items to be discussed at future meetings where there was a perceived conflict of interest involving her, Prof Peter Aggett will act as Chair.  The Chair emphasised the need for the Committee to be open and transparent in its assessment process and the importance of defining clear terms of reference for the review at the outset. The Chair also highlighted the importance of clear lines of reporting, and pointed to the potential of SACN’s website in providing public information on the progress of the review.  Agenda item 4 – Consultation on the Code of Practice for Scientific Advisory Committees (SACN/10/19)   - There should be a section on Committee independence and impartiality as these are important to the integrity of a Committees advice - There is no specific mention of observers from devolved administrations, lay members or industry and consumer representatives. - Members were unclear on the handling of the interests they declare | Members suggested moving relevant information on committee independence and impartiality forward from annex 2.  Conflicts of interest should be referred to as declarations of interest; and the section expanded to take on board both potential intellectual and financial conflicts.  Mention of observers from devolved administrations, lay members or industry and consumer representatives needs to be addressed in the committee’s role and remit section  Members requested that the brief document on how the SACN works be updated to explain handling of declared CoI and circulated to members for information. |
| 33 | 14/02/2011 | No changes to declarations of interest  It was noted that the (vitamin D) Working Group does not include a consumer representative.  Professor MacDonald will be attending Food Network (part of ‘responsibility deal’) on behalf of SACN. | Members were reminded that this would be rectified (consumer representative included in Vit D working group) following the conclusion of the current appointments process.  It was noted that the Committee were pleased to be represented at the Responsibility Deal meetings. The Chair recommended that a second representative should be identified in case Professor Macdonald is unavailable to attend any of the meetings. |
| 34 | 07/06/2011 | No changes to declarations of interest | None |
| 35 | 19/10/2011 | No changes to declarations of interest  Professor Macdonald informed members that the Responsibility Deal Food Network has made significant progress on the first three pledges: salt reduction, trans fats removal and out-of-home calorie labelling. | None |
| 36 | 10/02/2012 | No changes to declarations of interest | None |
| 37 | 12/06/2012 | No changes to declarations of interest | None |
| 38 | 06/02/2013 | No changes to declarations of interest | None |
| 39 | 12/06/2013 | Professor Peter Aggett declared that he had joined a European Food Safety Authority (EFSA) Food and Feeds Panel Working Group on Genetically Modified Organisms. | None |
| 40 | 09/10/2013 | No changes to declarations of interest  Members raised concerns regarding EFSA’s current investigation into a potential reduction of iodine in animal feed and the negative impact this would have on the iodine content of milk. This issue will need to be addressed should a risk assessment be carried out. Professor Aggett declared a conflict of interest as a member of the EFSA Committee on Animal Feed Stuffs. The Chair reported that the UK Advisory Committee on Animal Feeding stuffs (ACAF) has this issue on their agenda for its autumn meeting, and that it might be useful for the secretariats to communicate about potential joint discussions. | None |
| 41 | 26/02/2014 | Declarations of interest   - The Committee was updated on the recent media attention surrounding the declared interests of Carbohydrates Working Group members, highlighting that Public Health England (PHE) released a statement in support of the carbohydrates and health review, and both the PHE Chief Executive and PS (PH) spoke to Professor Ian Macdonald in support of him remaining on the Committee and as Chair of the Carbohydrates Working Group.  It was highlighted that the Chair of SACN is also supportive of the integrity of the Carbohydrates Working Group’s evaluation. - Professor Macdonald informed members that none of the companies have questioned him on the carbohydrates and health review, and clarified the current situation with regards to his declarations of interest as follows: He will not attend advisory board meetings at Coca Cola and Mars Europe at least until the carbohydrates and health review is completed; If he renews his involvement with the Coca Cola and Mars Europe Advisory Boards after the review is completed, any honorarium will be directed to his University; - Professor Macdonald also highlighted that he was approached by International Life Sciences Institute (ILSI) about being involved with the Dietary Carbohydrate Task force but also informed them that he did not wish to be part of this project until after the Carbohydrates and Health report has been finalised. - The Chair thanked members of the Working Group, who had received media attention, for their commitment to SACN. | Members discussed the processes in place to ensure the transparency and integrity of the review, listed in the statement by PHE and the Committee echoed their full support of the Working Group.  He will continue to have contact with The Mars Scientific Advisory Council, as this is based on his involvement with the Waltham Centre for Pet Nutrition, the honorarium for which goes to his University.  It was confirmed that the Committee will discuss SACN working practices at the main meeting in June 2014.  Given the lack of previous documentation of the chair of the carbohydrates working groups CoI and any subsequent diminishing action taken, in addition to the unclear wording here, it is not obvious that there were any efforts made to reduce the impact of a clear CoI before press attention.  Additionally, the review mentioned by the press was commissioned during the period considered by this analysis, but I McD has made no further CoI declarations nor has his participation with interested industries been discussed |
| 42 | 09/04/2014 | Dr David Mela noted that he had been requested to comment on the WHO draft sugar recommendations by his employer and trade groups and that he did so strictly without disclosing any details of SACN’s deliberations which were not already in the public domain.  Professor Ian Macdonald declared that he had declined to comment on the recently published WHO draft recommendations on sugar intake when approached by the media.  Comments attributed to him in recent media coverage originated from the media interviews he gave in January, and he had not made any further media statements since then.  Professor Julie Lovegrove informed Members that she is currently in negotiations with PepsiCo for research funding. |  |
| 43 | 04/06/2014 | changes to their declarations of interests; the following were declared: Professor Ian Macdonald – gave a talk in April at the European Hydration Institute, which focused on tea, milk and orange juice; and Dr Anthony Williams – GlaxoSmithKline shareholding.  Agenda item 4 – Declarations of interest (SACN/14/07)   - The Chair introduced the paper on declarations of interest, and asked for insight from Gemma Paramor, who has past experience of stringent compliance procedures in the fund management industry.  Members were informed that this role involved acting on behalf of retirees and other members of the public in investing their savings; therefore, it was important that personal interests were checked thoroughly. These procedures were applied to the individual employee and their partner and included quarterly self-certification of interests, an annual code of conduct questionnaire, rules for accepting hospitality and gifts, pre-clearance requirements for personal investment activity, insider trading policy and a compliance assessment as part of annual appraisals.  The annual code of conduct questionnaire collected information on the following areas: hospitality/gifts received; share ownership; external posts/responsibilities; personal relationships; and required a declaration of an annual read of compliance policy. - It was said that fund managers sought pre-clearance before making personal investments and would not trade in shares where they held insider information until it was public knowledge. In this context, some members informed the Committee that they decided not to give talks at events on vitamin D until the SACN Working Group on this subject had concluded. - It was said that it is the perception of some within the scientific community that SACN will automatically comply with decisions already made by PHE, and that the Committee should clarify its relationship to government as well as its independence. | The secretariat confirmed that members do seek their advice on whether to attend certain events and it was said that contributing to a SACN Working Group should not preclude members from presenting on their area of expertise, or restrict their ability to practise.  It is important for SACN members to be involved in discussions in the wider scientific  Community.  Members suggested that SACN:   - emphasise that SACN reviews are not about developing policy options; - define declared interests, and both actual and perceived conflicts; - consider whether to declare interests of partners or first degree relatives; - consider whether to declare gifts/hospitality of relevance to SACN work.   The Chair confirmed that the secretariat will take on board all these suggestions and update code of conduct documentation making SACN procedures for considering declarations of interest more transparent, which will also provide clearer guidance for members. |

Professor MacDonald reports to SACN on carbohydrates working group and updates from the responsibility deal food network, which he also attends meetings of. This is despite his known and declared interest (receiving research funds from and consulting) with corporations within the food industry.

It was unclear from the minutes of meeting 41 on 26/02/2014 whether these arrangements around Prof MacDonald’s perceived CoI were noted in previous minutes. All available minutes from the introduction of the carbohydrates working group were subsequently analysed, though no record was found.

Government statements on:

- SACN carbohydrate report following media coverage of potential CoI:

<https://www.gov.uk/government/news/uk-scientific-advisory-commission-on-nutrition-to-debate-recommended-sugar-levels>

- Code of practice for scientific committees

<https://www.gov.uk/government/uploads/system/uploads/attachment_data/file/278498/11-1382-code-of-practice-scientific-advisory-committees.pdf>
